# Chromosome-level reference genome for the Jonah crab, *Cancer borealis*

**DOI:** 10.1093/g3journal/jkae254

**Published:** 2024-11-06

**Authors:** Jennifer M Polinski, Timothy P O’Donnell, Andrea G Bodnar

**Affiliations:** Gloucester Marine Genomics Institute, Gloucester, MA 01930, USA; Gloucester Marine Genomics Institute, Gloucester, MA 01930, USA; Gloucester Marine Genomics Institute, Gloucester, MA 01930, USA

**Keywords:** Jonah crab, *Cancer borealis;* reference genome, de novo whole genome sequencing (WGS), fisheries, neuroscience

## Abstract

The Jonah crab, *Cancer borealis*, is integral to marine ecosystems and supports a rapidly growing commercial fishery in the northwest Atlantic Ocean. This species also has a long history as a model for neuroscience that has expanded our understanding of central pattern generators, neuromodulation, synaptic plasticity, and the connectivity of neural circuits. Here, we present a highly contiguous reference genome for the Jonah crab that will provide an essential resource to advance fisheries, conservation, and biomedical research. Using a combination of PacBio long-read sequencing and Omni-C scaffolding, we generated a final genome assembly spanning 691 Mb covering 51 chromosome-length scaffolds and 106 additional contigs. Benchmarking Universal Single-Copy Ortholog (BUSCO) analysis indicated a high-quality assembly with a completeness score of 90.8%. Repeat annotation identified 1,649 repeat families making up 48.27% of the Jonah crab genome. Gene model predictions annotated 24,830 protein coding genes with a 92.3% BUSCO score. Gene family evolution analysis revealed the expansion of gene families associated with nervous system function, and targeted analysis revealed an extensive repertoire of neural genes. The Jonah crab genome will not only provide a resource for neuroscience research but will also serve as a foundation to investigate adaptation to stress and population structure to support sustainable fisheries management during this time of rapidly changing environmental conditions in the northwest Atlantic Ocean.

## Introduction

Jonah crab (*Cancer borealis*) is a brachyuran crab species native to the Atlantic Ocean off the coast of North America from Nova Scotia, Canada to Florida, USA occupying the intertidal to depths of 800 m (Stehlik *et al*. 1991). Jonah crabs were historically considered bycatch of the lobster fishery, but declining lobster catches in southern New England and the development of new markets have increased the ex-vessel price of Jonah crab and caused harvesters to shift efforts to target Jonah crab (ASMFC 2023). Accordingly, Jonah crab landings have steadily increased since 1996, averaging 16 million pounds landed annually over the last decade (ACCSP 2024). The Jonah crab fishery has rapidly expanded at a time when the stock status is still unknown, and there has been limited science-based data available to support management, resulting in a high level of uncertainty regarding the impact of the fishery and the potential for overexploitation of this ecologically important benthic predator (ASMFC 2023). In response to potential overfishing, the Atlantic States Marine Fisheries Commission conducted the first Jonah crab stock assessment for US waters in 2023 with stock delineation as one of the primary research objectives. Understanding the genetic population structure of Jonah crab would contribute to delineating biologically relevant stock boundaries to complement the stock assessment and promote a sustainable and well-managed fishery.

Beyond the economic value offered by the Jonah crab fishery, this species is also a valuable model animal for understanding neural control of rhythmic behaviors, focusing on the cardiac and stomatogastric neural circuits. Using the Jonah crab as a model species has contributed to significant discoveries including understanding cardiac ganglion modulators (Cruz-Bermúdez and Marder 2007), factors that affect pyloric and gastric rhythm (Hamood *et al*. 2015; Kushinsky *et al*. 2019; Martinez *et al*. 2019), and the location and function of synapses (Rue *et al*. 2022). Jonah crab have also been used as a model to better understand the endocrine system including neuroendrocrine signaling (Christie 2011) and seasonal fluctuation in sex hormones (Lawrence *et al*. 2022). Researchers investigating these processes in Jonah crab have typically relied on a published neural transcriptome (Northcutt *et al*. 2016) and a reference genome of related species like the American lobster (Polinski *et al*. 2021) to conduct their analyses in the absence of a reference genome for Jonah crab.

In this study, we have assembled and annotated a chromosome-length reference genome for Jonah crab as a resource to advance studies of its biology, ecology, and population genetics. This genomic resource will enable advanced population genomics techniques such as low-coverage whole-genome sequencing (WGS), which requires a reference genome. Incorporating low-coverage WGS data for Jonah crab will contribute to the establishment of biologically accurate stock boundaries to promote sustainable management and can determine signals of local adaptation in response to changing environmental conditions. Additionally, the reference genome will enhance the utility of the Jonah crab as a model species for the biomedical field, allowing for the complete assessment of the genes involved in nervous system and endocrine function.

## Methods

### Biological sample collection

Jonah crabs used for this study were harvested from the Gulf of Maine in May 2021 and purchased from the Fresh Lobster Company (Gloucester, MA, USA). Five male Jonah crabs were maintained overnight at the Gloucester Marine Genomics Institute in an aquarium that contained filtered natural seawater maintained at 9°C. Several tissues (walking leg muscle, gonad, hemocytes, gill, and hepatopancreas) were dissected from the crabs and placed in individual 2-mL cryovials before being flash-frozen in liquid nitrogen and stored at −80°C until shipment. A male was selected for genome sequencing because, although the sex determination system of Jonah crabs is not known, closely related brachyuran species including *Portunus trituberculatus* have been shown to have male heterogametic (XX/XY) sex determination (Waiho *et al*. 2021). Frozen muscle and testis tissue from one individual male (carapace width = 175 mm) was shipped on dry ice to Dovetail Genomics for the reference genome. Additionally, hemocytes, muscle, gill, and hepatopancreas tissues, sampled from three individuals, were shipped in a similar manner to GENEWIZ (South Plainfield, NJ, USA) for RNA sequencing (RNAseq) analysis. Walking leg muscle tissue from the individual used to generate the reference genome (JC504) was archived in the Ocean Genome Legacy biorepository (https://ogl.northeastern.edu/) with the sample ID A53516 and specimen ID S36660 (Supplementary Table S1). Tissues (muscle, gill, hepatopancreas, heart, and testis) from three additional Jonah crabs were also archived in the Ocean Genome Legacy biorepository (Supplementary Table S1).

### Library preparation and sequencing

DNA extraction, library preparation, and Pacific Biosciences (PacBio; Menlo Park, CA, USA) sequencing were performed by Dovetail Genomics (Cantata Bio, LLC). For DNA extraction, 150 mg of testis tissue was used as input in a phenol/chloroform extraction. DNA was quantified with a Qubit 2.0 fluorometer, and PacBio SMRTbell libraries were constructed using a SMRTbell Express Template Prep Kit 2.0. Libraries were sequenced on PacBio Sequel II 8M SMRT cells, generating 344 Gbp of CLR data.

For Omni-C library preparation, chromatin was fixed in the muscle tissue with formaldehyde and then extracted. Fixed chromatin was digested with DNase I, ends were repaired and ligated to biotinylated bridge adapters, followed by proximity ligation of adapter-containing ends. Cross links were reversed prior to DNA purification. Biotin not internal to ligated fragments was removed, and sequencing libraries were generated using NEBNext Ultra enzymes (New England Biolabs, Ipswich, MA, USA) and Illumina-compatible adapters. Biotin-containing fragments were isolated with streptavidin beads. The library was PCR-enriched and sequenced on an Illumina HiSeqX. RNA from hemocytes, muscle, gill, and hepatopancreas tissues were extracted and sequenced by GENEWIZ (from Azenta Life Sciences) on an Illumina NovaSeq 6000.

### De novo genome assembly

PacBio sequencing generated a total of 22,144,746 CLR reads totaling 345.7 Gb, which were combined into a single fastq file. Trimmomatic (v0.38) was used to remove reads <10 kb in length (Bolger *et al*. 2014), resulting in 307.1 Gb (13.09 M reads) for assembly input. Multiple long-read assembly algorithms were tested, including WTDBG2, Flye, Canu, Shasta, and Miniasm. The preliminary assembly generated by Flye exhibited the best completeness score based on Benchmarking Universal Single Copy Ortholog (BUSCO) analysis with the arthopod_odb10 gene set (Kolmogorov *et al*. 2019; Manni *et al*. 2021). Flye was run in “pacbio-raw” mode, which takes into account the higher error rate present in CLR data. Assembly statistics for all assemblers tested can be found on the project GitHub repository (https://github.com/jmpolinski/Jonah-Crab-Genome/tree/main/2_preliminary-assembly).

Prior to scaffolding with Omni-C proximity ligation sequencing data, the Flye preliminary assembly was filtered to remove contigs <10 kb in length. Juicer (v1.6; https://github.com/aidenlab/juicer) was used to analyze Omni-C data with the filtered Flye assembly, and the “merged_nodups.txt” files output from Juicer was used as input for 3D-DNA (https://github.com/aidenlab/3d-dna) to generate a contact map (Durand *et al*. 2016; Dudchenko *et al*. 2017). The contact map was manually reviewed in Juicebox Assembly Tools visualization and analysis software (https://github.com/aidenlab/Juicebox). Reference mitochondrial genomes of two closely related species, *Cancer magister* (GenBank: MN371144.1) and *Cancer pagurus* (GenBank: MN334534.1) were used to identify and remove mitochondrial sequence from the assembly post-scaffolding.

Benchmarking Universal Single-Copy Orthologs (BUSCO) analysis was conducted to determine the completeness of the assembled reference genome. BUSCO was run in genome mode with the arthropod_odb10 gene set.

### Repeat annotation and masking

RepeatModeler (v2.0.4; https://www.repeatmasker.org/RepeatModeler/) was used to identify repetitive elements in the Jonah crab genome de novo (Smith and Hubley 2008). RepeatModeler employed the de novo repeat-finding programs RECON (v1.08) and RepeatScout (v1.0.6). The repeat library identified with RepeatModeler was used as input for RepeatMasker (v4.1.4; https://www.repeatmasker.org/RepeatMasker/) with RMBlast (v2.11.0) to mask repetitive elements in the genome sequence (Smit *et al*. 2013). The “-xsmall” setting was used to soft-mask the genome, outputting repeat regions in lower case and nonrepetitive sequence in capital letters.

### *Ab initio* gene model prediction and functional annotation

RNAseq data were mapped to the reference genome using HISAT2 (v2.2.1; https://daehwankimlab.github.io/hisat2/; Kim *et al*. 2019), and mapping results from all samples were combined into a single BAM file. Preliminary gene model prediction was performed on the soft-masked reference genome using BRAKER2 (v2.1.6; https://github.com/Gaius-Augustus/BRAKER; Gabriel *et al*. 2014) with Augustus (v3.4.0) and GeneMark-ES, using the combined RNAseq BAM file as evidence. Gene model prediction was redone when BRAKER3 (v3.0.3) was released, using the soft-masked reference genome and the hints file (hintsfile.gff) from the preliminary BRAKER2 run.

BRAKER3 produced 71,502 gene model predictions, which were then quality filtered. InterProScan (v5.63-95.0; https://interproscan-docs.readthedocs.io/en/latest/; Jones *et al*. 2014) identified 17,180 gene models with homology to references in the Pfam protein family database and InterPro (IPR) protein signature database and/or assigned a gene ontology (GO) term; these gene models were retained in the final gene set. Gene models not assigned Pfam, IPR, or GO terms were filtered to remove mono-exonic genes and queried against the Swiss-Prot and NR protein databases with blastp (-evalue 1e-10). Six thousand seven hundred eight genes showing homology to proteins in Swiss-Prot and an additional 4,913 with homology to references in NR were retained.

Functional annotation was performed using the Eukaryotic Non-Model Transcriptome Annotation Pipeline (EnTAP; v1.0.1; https://entap.readthedocs.io/en/latest/; Hart *et al*. 2020) with diamond (v2.1.8) and the invertebrate RefSeq (v2023-07; O'Leary *et al.* 2016) and Swiss-Prot protein databases (Gasteiger *et al*. 2001). In total, 3,971 isoforms were removed, retaining only the isoform with the highest homology to a reference hit or, for genes with no identified homologs, the longest isoform. The completeness of the final set of 24,830 gene models was checked with BUSCO (arthropoda_odb10, protein mode).

### Ortholog analysis and phylogenetic tree generation

The genome sequences and RefSeq gene feature files for all crustacean genomes with RefSeq annotations available at the time of analysis were downloaded from GenBank. gFACs (v1.1.2; https://gfacs.readthedocs.io/en/latest/) was used to extract protein models using the “-unique-genes-only” option to select one representative isoform for each gene (Caballero and Wegrzyn 2019). BUSCO was used to assess quality of each set, and only protein sets with >85% completeness and <10% duplication were retained for ortholog analysis. Thirteen crustacean species including the crabs *C. borealis*, *P. trituberculatus* (Tang *et al.* 2020; GCF_017591435.1)*, and Eriocheir sinensis* (Wang *et al.* 2022; GCF_024679095.1), the lobster *Homarus americanus* (Polinski *et al.* 2021; GCF_018991925.1), the crayfish *Procambarus clarkii* (Xu et al. 2021; GCF_020424385.1), the shrimps *Litopenaeus vannamei* (Zhang *et al.* 2019; GCF_003789085.1), *Penaeus chinensis* (Wang *et al*. 2022; GCF_019202785.1), and *Penaeus japonicus* (Kawato *et al*. 2021; GCF_017312705.1), the amphipod *Hyalella azteca* (Poynton *et al*. 2018; GCF_000764305.2), the copepods *Lepeophtheirus salmonis* (Joshi *et al*. 2022; GCF_016086655.3) and *Tigriopus californicus* (Barreto *et al*. 2018; GCF_007210705.1), the water fleas *Daphnia pulex* (Crease 1999; GCF_021134715.1) and *Daphnia magna* (Byeon *et al*. 2022; GCF_020631705.1) were used in ortholog analysis. Two insects *Drosophila melanogaster* (Hoskins *et al*. 2015; GCF_000001215.4) and *Bombus pyrosoma* were included as an outgroup. OrthoFinder (v2.5.4; https://github.com/davidemms/OrthoFinder) was run with default settings to identify groups of orthologous genes, or orthogroups, from the 15 protein sets and generated a preliminary species tree.

A time-scaled phylogenetic tree was generated using the TimeTree Wizard in MEGA11 (Tamura *et al*. 2021). Because MEGA11 uses a single branch for the outgroup, *D. melanogaster* was kept as the noncrustacean outgroup and *B. pyrosoma* was excluded from additional analyses. Sets of single-copy orthologs identified by OrthoFinder were separately aligned using muscle (v5.1; https://www.drive5.com/muscle/). Gblocks (v0.91b; https://home.cc.umanitoba.ca/∼psgendb/doc/Castresana/Gblocks_documentation.html) was used to extract conserved, well-aligned blocks from each alignment and concatenate them into a single protein alignment file. The MEGA11 TimeTree Wizard was used to generate a RelTime-ML tree, using the JTT model with all sites and uniform rates among sites. The following divergence times from timetree.org were used as calibration nodes: *C. borealis—P. trituberculatus* (max 225.0 MYA), *L. vannamei—P. chinensis* (57.8–108.3 MYA), *H. americanus—P. clarkii* (241.0–321.6 MYA), and *D. magna—T. californicus* (275–541 MYA; Kumar *et al*. 2022).

Orthogroup counts from OrthoFinder were reformatted, with *B. pyrosoma* counts removed, for use in computational analysis of gene family evolution (CAFE; v4.2.1). The time-scaled tree was used as input in 4 lambda parameters (see GitHub code for lambda tree). Significance cutoff was set to *P* < 0.01.

### Synteny analysis

Pairwise syntenic comparisons were inferred between *C. borealis* and *P. trituberculatus, C. borealis* and *E. sinensis*, and *P. trituberculatus* and *E. sinensis*. *P. trituberculatus* and *E. sinensis* were selected as the only other crab species with RefSeq gene annotations exhibiting BUSCO scores of >85% completeness at the time of analysis, as described for ortholog analysis. Only chromosome-length scaffolds from each genome assembly were included in this analysis, after it was confirmed that exclusion of short contigs did not negatively impact BUSCO completeness scores. Genome-wide alignments of coding regions and filtering of tandem duplications (tandem_Nmax = 10) and weak hits (cscore ≥0.7) were done with LAST (v1512; https://gitlab.com/mcfrith/last;  Kiełbasa *et al*. 2011). The MCscan python workflow (https://github.com/tanghaibao/jcvi/wiki/MCscan-(Python-version) was used for linkage clustering into syntenic blocks and for visualization (Tang *et al*. 2008).

## Results and discussion

### Reference genome assembly

**Fig. 1.**
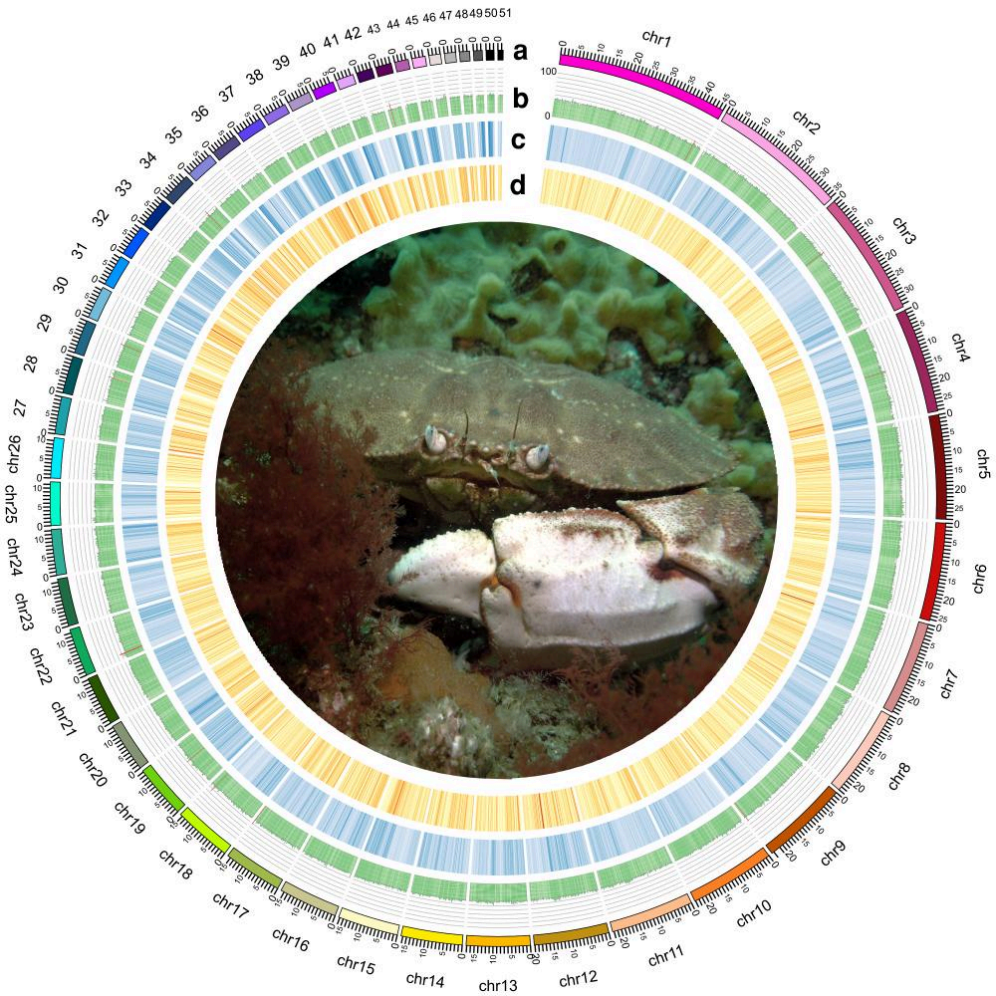
Circos plot of the Jonah crab (*C. borealis*) reference genome depicting a) the 51 chromosome-length scaffolds, b) percentage of guanine (G) and cytosine (C) bases (i.e. GC content; 0–100%) with regions exhibiting over 50% GC content depicted in red, c) repeat density with darker colors depicting higher density, and d) gene density with regions of low gene density in light yellow and higher density in red. A window size of 100 Kbp was used to calculate GC content, repeat density, and gene density. The central photograph depicts a Jonah crab in its natural habitat in MA, USA (Photo credit: J. M. Polinski).

A total of 13,090,793 PacBio long-reads (307.1 Gb) were used for de novo long-read assembly with Flye, and 904,493,702 Omni-C reads (135.6 Gb) were used in Juicer/3D-DNA scaffolding. The final chromosome-level genome assembly for the Jonah crab (*C. borealis*) consisted of 51 Omni-C scaffolds and 106 additional contigs, spanning a total of 691.19 Mb (Fig. 1 and Supplementary Fig. S1). The assembly exhibited a BUSCO completeness score of 90.8% (arthropoda_odb10, genome mode), indicating a high level of integrity, with additional genome assembly statistics listed in Table 1. At present, no karyotype exists for *C. borealis* or closely related species. However, the number of Omni-C scaffolds is within the range of those reported for other crab species with reference genomes, including *Scylla paramamosain* (*n* = 49) (Zhao *et al*. 2021), *Callinectes sapidus* (*n* = 50) (Bachvaroff *et al*. 2021), *P. trituberculatus* (*n* = 50) (Tang *et al*. 2020), and *E. sinensis* (*n* = 70) (Wang *et al*. 2022). Given this, the 51 Omni-C scaffolds are referred to as chromosomes hereafter.

**Table 1.** Cancer borealis genome assembly statistics.

Genome assembly	
Total scaffold	157
Total contigs	4655
Scaffold sequence total	691.19 Mbp
Contig sequence total	688.94 Mbp
Percent genome in gaps	0.325%
Scaffold N50/L50	17.182 Mb/13
Contig N50/L50	244.992 Mb/695
BUSCO (arthropod_obd10, genome mode)	
Complete (single/duplicated)	90.8% (87.4%/3.4%)
Fragmented	3.2%
Missing	6.0%
Gene models	
Number of genes	24,830
Mean exons/gene	6.8
Mean gene length	8,183 bp
Mean CDS length	1,486 bp
BUSCO [arthropod_obd10, protein mode]	
Complete (single/duplicated)	92.3% (88.1%/4.2%)
Fragmented	1.8%
Missing	5.9%

### Repeat and gene annotation

RepeatModeler and RepeatMasker identified 1,649 repeat families making up 48.48% of the *C. borealis* genome (333.66 Mb), dispersed across the genome (Fig. 1). Of these, 1,091 were classified as unknown (12.62% of genome). Simple repeats were the most common, making up 19.68% of the genome (Supplementary Table S2). Retroelements made up 12.68% of the genome, including long interspersed nuclear elements (9.16%), long terminal repeats (3.30%), and short interspersed nuclear elements (0.22%). DNA transposons made up 1.70%, small RNA 0.50%, satellites 0.01%, and low complexity repeats 1.29% (Supplementary Table S2).

*Ab initio* gene model predictions were quality filtered to remove low quality models, and isoforms were removed to produce a gene model set consisting of 24,830 protein coding genes for downstream analyses (Supplementary Table S3). BUSCO analysis with the arthropod (odb10) gene set in protein mode indicated the final gene set is 92.3% complete, with 1.8% fragmented and 5.9% missing (Table 1). Homology searches against the SwissProt database assigned functional annotations to 11,505 genes. Homology searches against the RefSeq invertebrate database identified 13,536 genes with homology to those in the database, although 3,425 of the references were annotated as uncharacterized proteins. Overall, 9,122 gene predictions had no homology to proteins in the RefSeq invertebrate or SwissProt databases. Gene model functional annotation results are available in Supplementary Table S3.

### Orthology and phylogeny

**Fig. 2.**
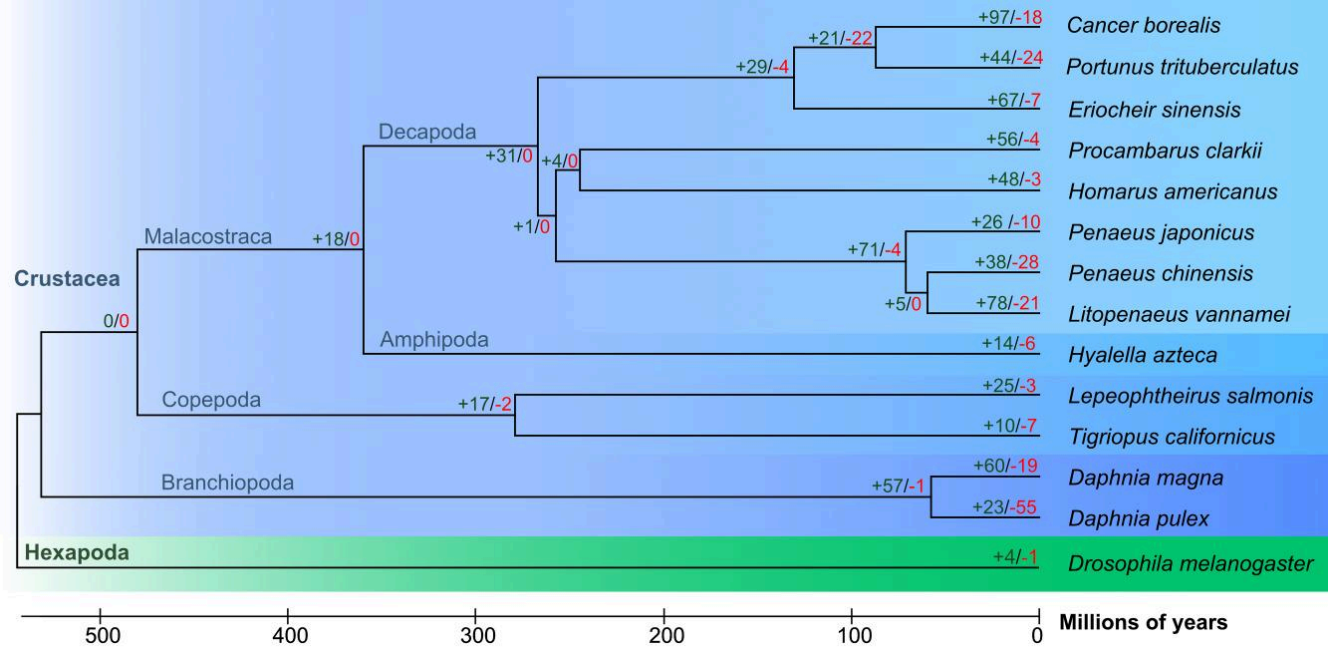
Phylogenetic tree of Crustaceans, with *D. melanogaster* as an arthropod outgroup. Branch lengths represent divergence time in millions of years ago. Number of rapidly evolving (*P* < 0.01) gene families identified with CAFE are shown at each Branch. Rapidly expanding gene families are in green font, and contracting are shown in red font.

Thirteen crustacean species were used in ortholog analysis, including the crabs *C. borealis*, *P. trituberculatus, and E. sinensis,* the lobster *H. americanus*, the crayfish *P. clarkii*, the shrimps *L. vannamei*, *P. chinensis*, and *P. japonicus*, the amphipod *H. azteca*, the copepods *L. salmonis* and *T. californicus*, the water fleas *D. pulex* and *D. magna*. *D. melanogaster* was included as an outgroup. A total of 22,546 orthogroups were identified by OrthoFinder analysis. The orthogroup set included 1,127 single-copy orthologs used to generate a time-scaled phylogenetic tree (Fig. 2; Supplementary Fig. S2). TimeTree analysis indicates that *C. borealis* diverged from *P. trituberculatus* ∼86.93 million years ago (MYA), and that the branch containing *C. borealis* and *P. trituberculatus* diverged from *E. sinensis* approximately 130.41 MYA (Fig. 2; Supplementary Fig. S2). Previous analysis by Cui *et al*. (2021) and Tang *et al*. (2020) indicated a slightly longer divergence times between *P. trituberculatus* and *E. sinensis* of 147.5 and 183.5 MYA, respectively, although both analyses did not include *C. borealis* or any other closely related species. The three included crab species diverged from other included decapod crustaceans ∼267.13 MYA (Fig. 2;  Supplementary Fig. S2).

The 22,546 orthogroups were used as input for CAFE analysis to identify rapidly evolving gene families among our selected crustacean species. In the lineage giving rise to *C. borealis*, there were 97 expanded and 18 contracted gene families (*P* < 0.01; Fig. 2; Supplementary Table S4). Of the 97 expanded gene families, 64 were annotated as uncharacterized and 9 had no match when subjected to BLAST searches indicating that much remains unknown about the biology of this animal. Three of the expanded gene families play a role in neuronal cell function (ionotropic glutamate receptors, syntaxin-binding protein 5-like, and the transcription factor Adf-1; Supplementary Table S4). Given the importance of *C. borealis* as a model for neuroscience research, we conducted a targeted examination of the neural gene complement. This analysis identified more than 1,200 gene predictions encoding neural-related proteins including 374 ligand-gated ion channels, 211 ion channels, 244 G protein-coupled receptors, and 404 other neuronal genes including those involved in regulating synapses, axon guidance, and neural development (Supplementary Table S5).

The 18 contracted gene families included 9 gene families annotated as uncharacterized or for which genes within the family did not share a common annotation. Orthogroups containing genes annotated as histones H2A, H2B, H3, and H4 were identified as contracted gene families, with 1, 0, 2, and 0 orthologs identified in *C. borealis*, respectively. However, further examination identified six gene models with homology to histone H3 that were included in other orthogroups. Further, homology searches of reference sequences against the genome identified 77 copies of histone H2A, 71 histone H2B, 8 additional histone H3 genes, and 5 copies of histone H4 (Supplementary Table S6). The reduced number of histone gene predictions in the original gene model set is likely a consequence of the small size of histone genes rather than a more general problem with this analysis. While *ab initio* gene prediction pipelines like BRAKER3 and analysis programs like CAFE provide fast and informative annotations and results, the suggestion of contracted histone gene families due to incomplete gene predictions exemplifies the need to manually confirm bioinformatically derived results.

### Syntenic alignments between crab genomes

**Fig. 3.**
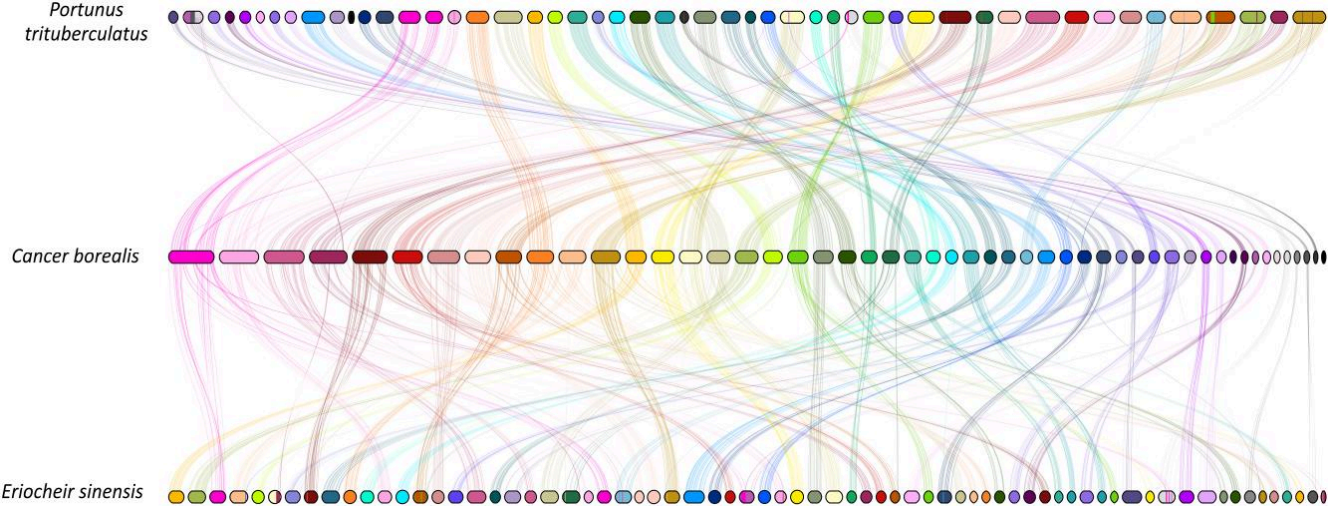
Pairwise syntenic alignments of chromosomes from *C. borealis* to *P. trituberculatus* and *E. sinensis*. Vertical lines connect orthologous gene blocks; colored based on the *C. borealis* chromosomes.

Pairwise syntenic alignments were conducted to evaluate chromosome organization and the conservation of homologous gene order among the crab species; the Jonah crab (*C. borealis)*, the swimming crab (*P. trituberculatus*), and the Chinese mitten crab (*E. sinensis*; Fig. 3). Consistent with their evolutionary relationships, there was a higher degree of conservation between *C. borealis* and *P. trituberculatus* with 32 chromosomes showing 1:1 synteny. In contrast, only 15 chromosomes share 1:1 synteny between *C. borealis* and *E. sinensis*. Interestingly, three chromosomes (42, 46, and 51) appear to be unique to *C. borealis* with no synteny with either of the other species, although this could be due to inclusion of only chromosome-length scaffolds in synteny analysis. These differences suggest dynamic evolutionary processes acting upon these genomes; however, the quality of genome assemblies is critical for reliable synteny analysis. A recent study demonstrated that a minimum N50 of 1 Mb is required for robust downstream synteny analysis (Liu *et al*. 2018). *C. borealis* and *P. trituberculatus* meet this criterion with N50 values of 17.18 Mb and 4.12 Mb, respectively. However, the N50 value for the *E. sinensis* genome assembly is 224 kb suggesting that some of the observed differences may be the results of lower contiguity in the other crustacean genome assemblies and potential assembly errors.

## Conclusion

This reference genome for Jonah crab is the most complete and contiguous reference for the species available to date. It will not only enhance the value of this historic model for neuroscience but will also advance research related to its biology, ecology, and fisheries management. The reference genome will enable high-resolution population genetic assessment using low-coverage WGS throughout the species range, which will provide insight into the connectivity of Jonah crab populations and identify key boundaries appropriate for management unit delineations. In addition, genomic data provide a foundation to increase our understanding of the genetic underpinnings of Jonah crab life history and biology and its vulnerability or resilience to environmental change. This is especially critical in this time of rapidly warming conditions in the northwest Atlantic Ocean that are already having a dramatic impact on the distribution and disease occurrence in other economically important crustaceans like the American lobster (*H. americanus*; Glenn and Pugh 2006; Pershing *et al*. 2015; Le Bris *et al*. 2018; McLean *et al*. 2018). Ultimately, these data will be instrumental for the establishment of a sustainable, well-managed fishery.

## Supplementary Material

jkae254_Supplementary_Data

## Data Availability

This Whole Genome Shotgun project has been deposited at DDBJ/ENA/GenBank under the accession JBCDWE000000000. The version described in this paper is version JBCDWE010000000. Data generated for this study is available under NCBI BioProject PRJNA1097536. PacBio CLR data and Omni-C data used for the genome assembly are available on the NCBI Sequencing Read Archive under accessions SRR28587252 and SRR28587251, respectively. RNA-seq Illumina data are available via SRA accessions SRR28587243-SRR28587250 and SRR28587253-SRR28587256. Information on all pipelines used for assembly and analysis, as well as the commands used to generate results, can be found on the project GitHub: https://github.com/jmpolinski/Jonah-Crab-Genome. Files related to gene model prediction and annotation, as well as files associated with RepeatModeler, phylogenetic tree generation, and synteny analysis can be found on Zenodo (doi.org/10.5281/zenodo.12746651). Supplemental material available at G3 online.
