# Supplementary material for: Chromosome-level reference genome for the Jonah crab, Cancer borealis
Source: G3 (Bethesda). 2024 Nov 6;15(1):jkae254. doi: 10.1093/g3journal/jkae254 (PMC11708212; doi:10.1093/g3journal/jkae254)
Supplement: jkae254_Supplementary_Data [file jkae254_supplementary_data.zip › Supplemental_Figures_and_Tables_S1-S2_G3-2024-405283.pdf]

SUPPLEMENTAL MATERIALS

Chromosome-level reference genome for the Jonah crab, *Cancer borealis*

Jennifer M. Polinski, Timothy P. O'Donnell, Andrea G. Bodnar

Gloucester Marine Genomics Institute, 417 Main Street, Gloucester, MA, USA 01930

Corresponding author: Jennifer M. Polinski (jennifer.polinski@gmgi.org)

Contents

Figure S1. Hi-C contact map

Figure S2. Time-scaled phylogenetic tree

Table S1. Accession IDs for archived Jonah crab tissue samples

Table S2. Repetitive element composition in the Jonah crab genome

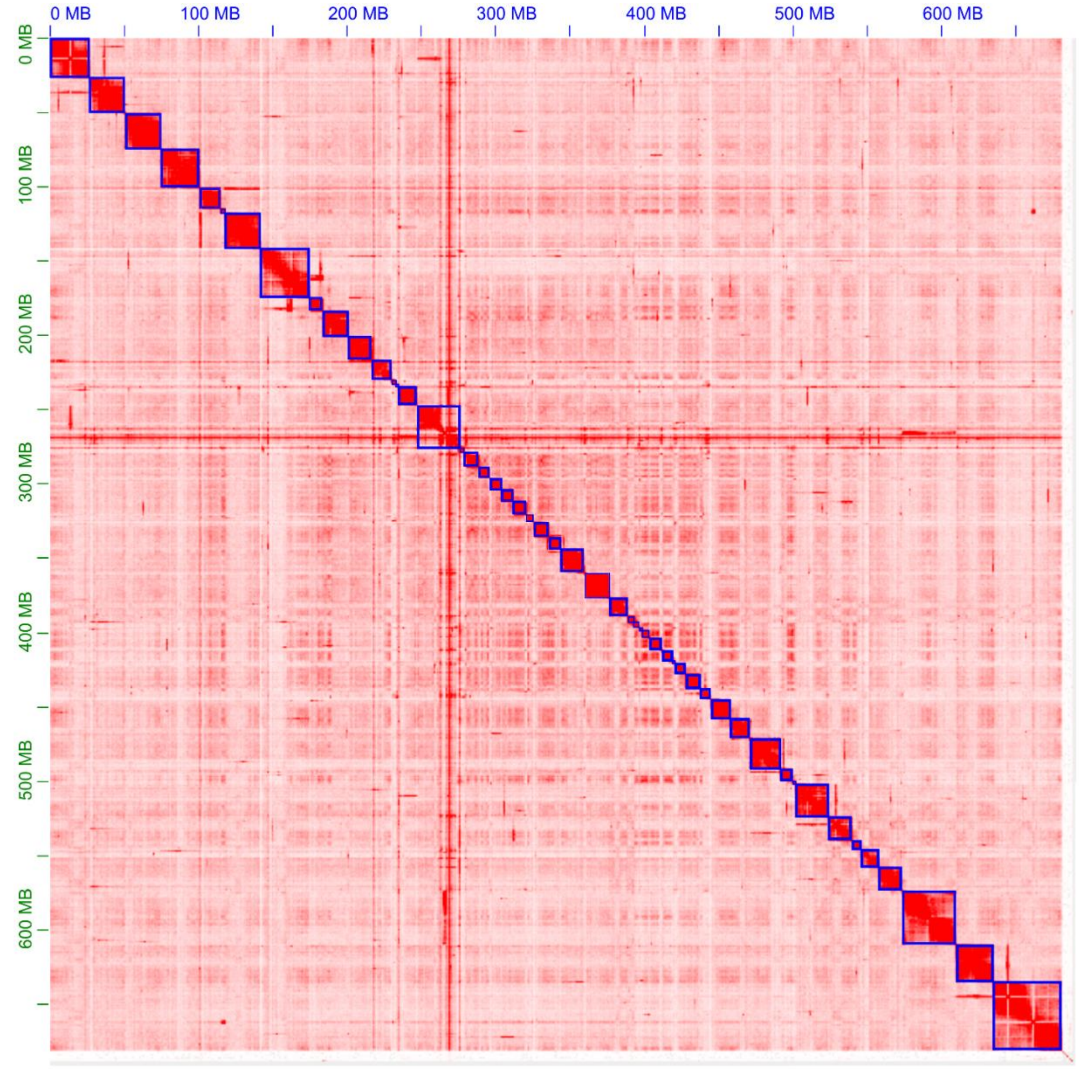

Figure S1. Contact map from Omni-C proximity ligation data, showing 51 chromosome-length scaffolds.

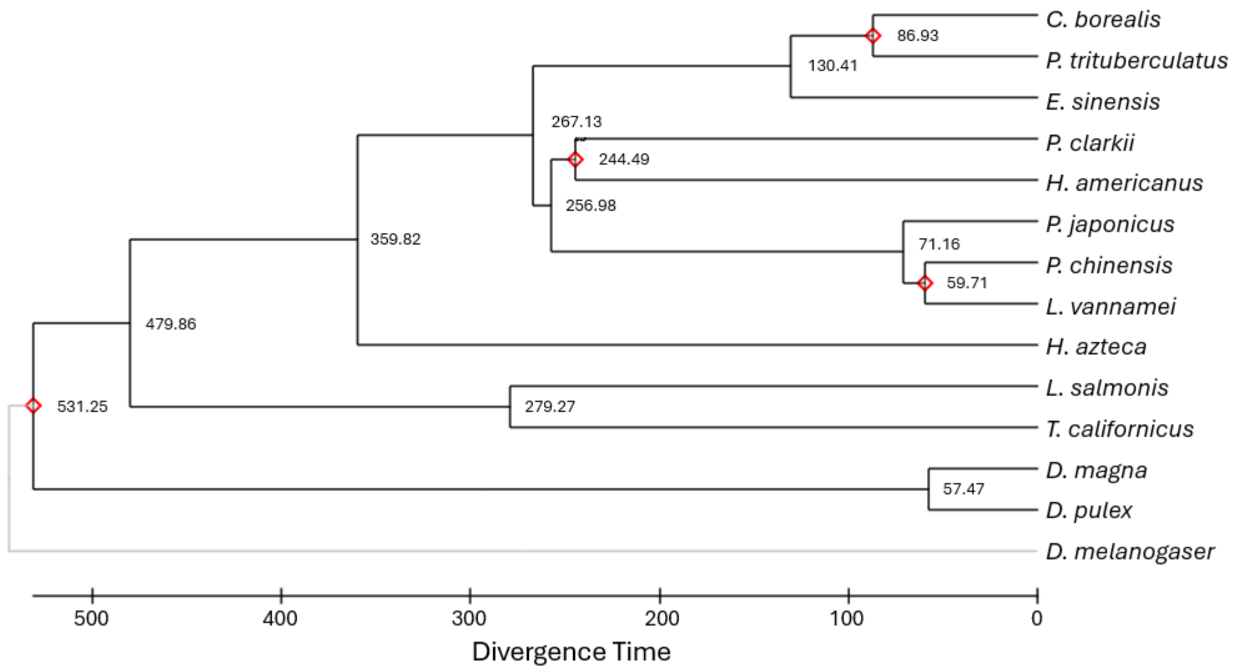

Figure S2. Time-scaled phylogenetic tree, generated with the MEGA11 TimeTree Wizard, with divergence time estimates shown at branch nodes. *D. melanogaster* was used as the outgroup.

Table S1. Accession IDs for the Jonah crab tissue samples archived in the Ocean Genome Legacy biorepository (https://ogl.northeastern.edu/)

Ocean Genome Legacy Accession IDs			
**Sample ID**	**Specimen ID**	**external ID**	**Tissue Type**
A53516	S36660	JC504	Muscle from walking leg
A53517	S36661	JC1	Gill
A53518	S36661	JC1	Hepatopancreas
A53519	S36661	JC1	Heart
A53520	S36661	JC1	Testis
A53521	S36661	JC1	Muscle from walking leg
A53522	S36662	JC2	Gill
A53523	S36662	JC2	Hepatopancreas
A53524	S36662	JC2	Heart
A53525	S36662	JC2	Testis
A53526	S36662	JC2	Muscle from walking leg
A53527	S36663	JC3	Gill
A53528	S36663	JC3	Hepatopancreas
A53529	S36663	JC3	Heart
A53530	S36663	JC3	Testis
A53531	S36663	JC3	Muscle from walking leg

Table S2. Repetitive element composition in the Jonah crab genome.

	Number of elements	Length occupied (bp)	Percentage of sequence
**Retroelements**	**206702**	**87655716**	**12.68%**
SINEs	***9420***	***1535497***	***0.22%***
Penelope	***3417***	***356205***	***0.05%***
LINEs:	***178531***	***63305802***	***9.16%***
CRE/SLACS	4256	1688564	0.24%
L2/CR1/Rex	90937	32145381	4.65%
R1/LOA/Jockey	17068	10400352	1.50%
R2/R4/NeSL	669	492864	0.07%
RTE/Bov-B	32888	7711610	1.12%
L1/CIN4	608	285467	0.04%
LTR elements:	***18751***	***22814417***	***3.30%***
BEL/Pao	677	1292175	0.19%
Ty1/Copia	86	246774	0.04%
Gypsy/DIRS1	17769	21224962	3.07%
Retroviral	0	0	0.00%
**DNA transposons**	**45862**	**11750688**	**1.70%**
hobo-Activator	8550	2179628	0.32%
Tc1-IS630-Pogo	23414	5352146	0.77%
En-Spm	0	0	0.00%
MULE-MuDR	1301	226568	0.03%
PiggyBac	336	166619	0.02%
Tourist/Harbinger	4099	1412589	0.20%
Other	528	229087	0.03%
**Rolling-circles**	**226**	**13869**	**0.00%**
**Unclassified**	**451665**	**87207921**	**12.62%**
**Small RNA**	**17707**	**3464046**	**0.50%**
**Satellites**	**385**	**63464**	**0.01%**
**Simple repeats**	**1251943**	**136041152**	**19.68%**
**Low complexity**	**112184**	**8950373**	**1.29%**
